# Pleural Management Pathways and Outcomes in Haematological Malignancy-Associated Malignant Pleural Effusion

**DOI:** 10.3390/jcm15135201

**Published:** 2026-07-03

**Authors:** Olivia Walsh, Alguili Elshekih, Dinesh Addala, Malivka Bhatnagar, Graham Collins, Eihab O. Bedawi, Najib Rahman

**Affiliations:** 1 Oxford Centre for Respiratory Medicine, Churchill Hospital, Oxford University Hospitals NHS Foundation Trust, Oxford OX3 9DU, UK; alguili.elsheikh@ouh.nhs.uk (A.E.); dinesh.addala@ouh.nhs.uk (D.A.); malvika.bhatnagar@ouh.nhs.uk (M.B.); eihab.bedawi@ouh.nhs.uk (E.O.B.); najib.rahman@ouh.nhs.uk (N.R.); 2 Oxford Pleural Unit, Oxford University Hospitals, Oxford OX3 9DU, UK; 3 Oxford Centre for Haematological Medicine, Churchill Hospital, Oxford University Hospitals NHS Foundation Trust, Oxford OX3 9DU, UK; graham.collins@ouh.nhs.uk

**Keywords:** pleural effusion, malignant pleural effusion, haemtatological malignancy

## Abstract

**Background**: Malignant pleural effusion (MPE) is a common complication of advanced malignancy associated with significant symptom burden. Current British Thoracic Society guidance supports early definitive pleural intervention for recurrent MPE; however, these recommendations are largely derived from solid tumour populations, and their applicability to haematological malignancy remains uncertain. **Methods**: We performed a retrospective cohort study of adult patients with cytology-confirmed haematological malignancy-associated MPE managed by the Oxford Pleural Unit between 2015 and 2025. Consecutive patients undergoing at least one pleural procedure were included. Definitive pleural intervention was defined as indwelling pleural catheter (IPC) insertion, talc pleurodesis, or surgical pleural intervention. The primary outcome was time from first pleural procedure to definitive pleural intervention. Cox proportional hazards regression was used to explore factors associated with progression to definitive intervention. **Results**: Among 910 patients with cytology-positive MPE, 116 (12.7%) had underlying haematological malignancy. Initial pleural management was therapeutic aspiration in 90 patients (78%), chest drain insertion in 24 (21%), and direct IPC insertion in 2 (2%). Among patients managed with an aspiration-first strategy, 63/90 (70%) avoided definitive pleural intervention entirely. Thirty-six patients (40%) required only a single aspiration before systemic therapy controlled the effusion, while 27 (30%) were managed with repeat aspirations alone. Overall, only 35/116 (30%) patients required definitive pleural intervention during follow-up. Non-chemo-responsive disease was associated with earlier progression to definitive pleural intervention (adjusted HR 1.85, 95% CI 1.10–3.10; *p* = 0.02). **Conclusions**: In contrast to solid tumour-associated MPE, many patients with haematological malignancy-associated MPE can be successfully managed without early definitive pleural intervention. An aspiration-first strategy may therefore be appropriate in selected patients with anticipated chemo-responsive disease.

## 1. Introduction

Malignant pleural effusion (MPE) is a common complication of advanced malignancy and is associated with substantial symptom burden, impaired quality of life, and recurrent healthcare utilisation. Current management strategies for MPE focus on symptom control and reduction in recurrent pleural fluid accumulation through definitive pleural intervention, most commonly indwelling pleural catheter (IPC) insertion or talc pleurodesis. Contemporary British Thoracic Society (BTS) guidance recommends a patient-centred approach to MPE management, with early consideration of definitive pleural intervention rather than repeated therapeutic aspiration alone [1].

However, the evidence underpinning current pleural management pathways is derived predominantly from studies involving solid tumour malignancies, including landmark randomised trials such as TIME-2 and AMPLE [2,3]. In these populations, pleural effusions frequently recur following initial aspiration, and definitive pleural intervention is commonly required to achieve sustained symptom control. Consequently, repeated therapeutic aspiration is generally regarded as a temporising rather than definitive strategy in solid tumour-associated MPE.

Whether these management paradigms are directly applicable to haematological malignancy-associated MPE remains uncertain. In contrast to many advanced solid tumours, haematological malignancies such as lymphoma and plasma cell dyscrasias may demonstrate substantial responsiveness to systemic anti-cancer therapy, potentially allowing resolution of pleural effusions without the need for definitive pleural intervention. Previous studies have described the occurrence and prognostic implications of pleural effusions in lymphoma populations [4,5,6], but contemporary data examining pleural management trajectories, procedural requirements, and outcomes in haematological malignancy-associated MPE remain limited, particularly within specialist pleural services.

Improved understanding of pleural management pathways in this population is clinically important. Routine extrapolation of solid tumour-derived MPE strategies may risk over-intervention in patients whose pleural effusions may resolve following systemic therapy alone, while delayed definitive pleural intervention in refractory disease may contribute to ongoing symptom burden and repeated procedures.

We therefore performed a retrospective cohort study of patients with cytology-confirmed haematological malignancy-associated MPE managed within a specialist pleural service, aiming to characterise pleural management trajectories, rates of definitive pleural intervention, and factors associated with progression to definitive pleural management.

## 2. Materials and Methods

### 2.1. Study Design and Population

We performed a retrospective cohort study of adult patients with cytology-confirmed malignant pleural effusion (MPE) secondary to haematological malignancy managed by the Oxford Pleural Unit between January 2015 and January 2025. Consecutive patients undergoing at least one pleural procedure for symptomatic pleural effusion were included. Patients with non-malignant pleural effusions or without cytological confirmation of malignant pleural involvement were excluded.

### 2.2. Data Collection

Clinical data were collected retrospectively from electronic medical records, pleural procedure databases, imaging reports, and multidisciplinary team documentation. Baseline demographic data, underlying haematological malignancy subtype, pleural intervention details, systemic anti-cancer therapy, imaging findings, and clinical outcomes were recorded.

Haematological malignancies were categorised into lymphoma, plasma cell dyscrasia, leukaemia, and other haematological subtypes. Chemo-responsiveness was determined retrospectively from multidisciplinary team documentation, clinic correspondence, and follow-up imaging. Disease was classified as chemo-responsive where systemic anti-cancer therapy resulted in documented reduction or resolution of pleural effusion, improvement in pleural disease burden, and/or avoidance of further pleural intervention within 3 months of treatment initiation. Formal response criteria such as Lugano or IMWG criteria were not routinely available and were therefore not used.

Septated or complex pleural effusion was defined according to thoracic ultrasound or computed tomography findings demonstrating pleural septations, loculation, or complex pleural fluid characteristics.

### 2.3. Pleural Management Pathways

Initial pleural management decisions were made by the treating pleural physician according to routine practice within a specialist pleural service. Therapeutic aspiration was commonly performed as a first-line intervention to assess symptomatic response and evaluate lung re-expansion. Definitive pleural intervention was defined as indwelling pleural catheter (IPC) insertion, talc pleurodesis, or surgical pleural intervention. Repeat therapeutic aspiration without progression to IPC or pleurodesis was classified as non-definitive pleural management.

### 2.4. Outcomes

The primary outcome was time from first pleural procedure to definitive pleural intervention. Secondary outcomes included rates of aspiration-only management, requirement for repeat pleural procedures, and factors associated with progression to definitive pleural intervention.

### 2.5. Statistical Analysis

Continuous variables are presented as median and interquartile range (IQR), while categorical variables are presented as frequency and percentage. Associations with time to definitive pleural intervention were explored using Cox proportional hazards regression and are reported as hazard ratios (HRs) with 95% confidence intervals (CIs). Variables considered clinically relevant, including age, haematological malignancy subtype, chemo-responsiveness, and septated or complex pleural effusion, were evaluated in exploratory univariable analyses. An exploratory multivariable model adjusting for age and haematological subtype was subsequently performed. Given the retrospective observational design and limited event number, all regression analyses were considered exploratory and hypothesis-generating. Statistical significance was defined as *p* < 0.05. Given the limited number of definitive pleural intervention events (*n* = 35), multivariable modelling was deliberately restricted to a small number of clinically relevant covariates to minimise model overfitting. Age and haematological malignancy subtype were selected a priori as potential confounders based on clinical plausibility.

## 3. Results

### 3.1. Cohort Characteristics

Between 2015 and 2025, 910 patients with cytology-confirmed malignant pleural effusion (MPE) were managed by the Oxford Pleural Unit, of whom 116 (12.7%) had underlying haematological malignancy. Median age was 68 years (IQR 60–76). Lymphoma represented the most common underlying diagnosis (approximately 60%), followed by plasma cell dyscrasias and leukaemia.

Initial pleural management consisted of therapeutic aspiration in 90 patients (78%), chest drain insertion in 24 (21%), and direct indwelling pleural catheter (IPC) insertion in 2 patients (2%).

### 3.2. Pleural Management Trajectories

Among patients initially managed with an aspiration-first strategy, 63/90 (70%) avoided definitive pleural intervention entirely during follow-up. In total, 36 patients (40%) required only a single therapeutic aspiration prior to resolution of the effusion following systemic anti-cancer therapy, while a further 27 patients (30%) were managed with repeat aspirations alone without progression to IPC insertion or pleurodesis (Table 1).

Overall, only 35/116 patients (30%) required definitive pleural intervention at any point during follow-up. This contrasts with solid tumour-associated MPE, in which recurrence following therapeutic aspiration is common and approximately 70–80% of patients progress to definitive pleural intervention in randomised studies such as TIME-2.2.

Patients initially managed with chest drain insertion infrequently required further pleural procedures, while those undergoing direct IPC insertion did not require additional pleural intervention during follow-up.

### 3.3. Factors Associated with Progression to Definitive Pleural Intervention

In univariable Cox proportional hazards analysis, non-chemo-responsive disease was associated with earlier progression to definitive pleural intervention (hazard ratio [HR] 2.10, 95% confidence interval [CI] 1.30–3.40; *p* = 0.003). Septated or complex pleural effusion was also associated with earlier progression to definitive intervention (HR 1.70, 95% CI 1.02–2.85; *p* = 0.04).

In contrast, haematological malignancy subtype (lymphoma versus other subtypes) and age were not significantly associated with progression to definitive pleural intervention (HR 1.45, 95% CI 0.90–2.35; *p* = 0.12 and HR 1.08 per 10-year increase, 95% CI 0.92–1.27; *p* = 0.35, respectively).

In an exploratory multivariable Cox model adjusting for age and haematological malignancy subtype, non-chemo-responsive disease remained independently associated with earlier progression to definitive pleural intervention (adjusted HR 1.85, 95% CI 1.10–3.10; *p* = 0.02).

Septated or complex pleural effusions may represent a marker of greater pleural organisation and disease burden, potentially reducing the likelihood of resolution with systemic therapy alone.

## 4. Discussion

**Figure 1:**
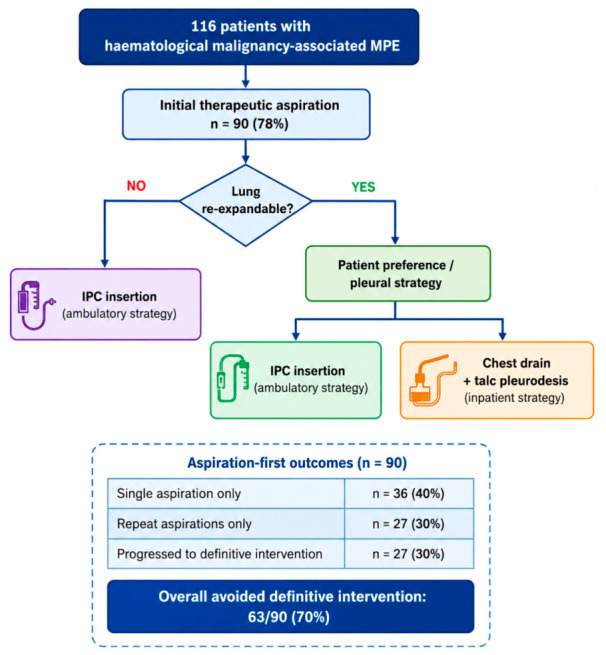
Pleural management pathways and outcomes in haematological malignancy-associated malignant pleural effusion. Flow diagram demonstrating pleural management trajectories among patients with haematological malignancy-associated malignant pleural effusion (MPE) managed with an aspiration-first strategy. Following initial therapeutic aspiration, subsequent pleural management was guided by lung re-expansion, patient preference, and anticipated disease responsiveness. Among patients initially managed with therapeutic aspiration, 63/90 (70%) avoided definitive pleural intervention entirely, including 36 patients managed with a single aspiration only and 27 managed with repeat aspirations alone. Overall, only 27/90 (30%) progressed to definitive pleural intervention with either indwelling pleural catheter (IPC) insertion or chest drain insertion with talc pleurodesis.

In this retrospective cohort study of patients with haematological malignancy-associated malignant pleural effusion (MPE) managed within a specialist pleural service, the majority of patients initially managed with an aspiration-first strategy avoided definitive pleural intervention entirely. As demonstrated in Figure 1, among patients undergoing initial therapeutic aspiration, 70% did not progress to indwelling pleural catheter (IPC) insertion or pleurodesis during follow-up, with many requiring only a single pleural procedure prior to control of the effusion following systemic therapy. These findings contrast with established management paradigms in solid tumour-associated MPE, where recurrence following aspiration is common and definitive pleural intervention is frequently required [2,3].

Current British Thoracic Society (BTS) guidance recommends a patient-centred approach to MPE management, including early consideration of definitive pleural intervention [1]. However, the evidence underpinning these recommendations is derived predominantly from studies involving solid tumours, including trials such as TIME-2 and AMPLE, in which recurrent pleural fluid accumulation following aspiration is common [2,3]. In contrast, our findings suggest that a substantial proportion of patients with haematological malignancy-associated MPE may be managed successfully without early definitive pleural intervention, likely reflecting the chemo-responsive nature of many haematological malignancies.

Importantly, this difference is unlikely to be explained solely by younger age or improved physiological reserve. The median age of patients within our cohort was comparable to that reported in major solid tumour MPE studies, suggesting that underlying tumour biology and responsiveness to systemic therapy are more likely to account for the divergent pleural outcomes observed. Routine extrapolation of solid tumour-derived pleural management strategies may therefore risk over-intervention in selected patients with haematological malignancy-associated MPE.

**Figure 2:**
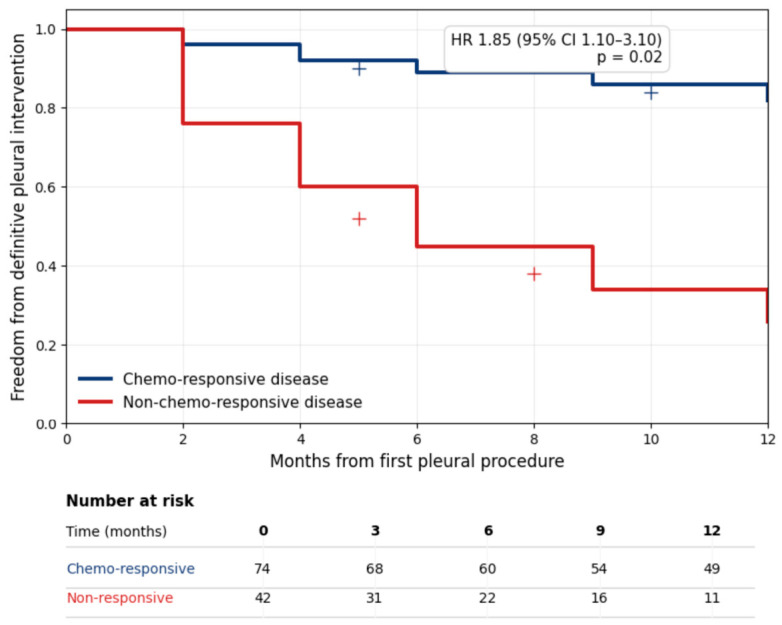
Time to definitive pleural intervention according to chemo-responsiveness. Kaplan–Meier analysis demonstrating freedom from definitive pleural intervention among patients with haematological malignancy-associated malignant pleural effusion stratified by chemo-responsiveness. Non-chemo-responsive disease was associated with earlier progression to definitive pleural intervention.

Non-chemo-responsive disease was associated with earlier progression to definitive pleural intervention, even following adjustment for age and haematological malignancy subtype. Similarly, septated or complex pleural effusions were associated with earlier escalation of pleural management. As shown in Figure 2, patients with non-chemo-responsive disease demonstrated substantially shorter freedom from definitive pleural intervention over time compared with patients responding to systemic therapy. These findings are clinically plausible and may reflect greater pleural organisation, higher pleural disease burden, and reduced likelihood of resolution with systemic therapy alone. Identification of patients unlikely to respond to systemic therapy may therefore be important in guiding earlier definitive pleural management.

Our findings do not contradict current BTS guidance but instead highlight an important subgroup in whom an initial non-definitive pleural strategy may be appropriate. An aspiration-first approach may allow symptom control while avoiding unnecessary pleural procedures in patients with anticipated chemo-responsive disease. Conversely, a distinct subgroup progresses rapidly to definitive pleural intervention, underscoring the need for improved risk stratification within this population.

This study has several limitations. Its retrospective single-centre design introduces potential selection bias and limits generalisability. Pleural management decisions were made according to clinician judgement within routine practice and may have evolved over the study period alongside advances in systemic anti-cancer therapy and pleural intervention pathways. Additionally, the regression analyses were exploratory and hypothesis-generating given the limited event number.

Furthermore, chemo-responsiveness was determined following initiation of systemic therapy and therefore represents a post-baseline variable. Consequently, the observed association between chemo-responsiveness and progression to definitive pleural intervention may be susceptible to immortal time bias and should be interpreted cautiously.

Nonetheless, this study represents one of the largest dedicated cohorts examining pleural management trajectories in haematological malignancy-associated MPE within a specialist pleural service. These findings support a more individualised approach to pleural management in this under-studied population and provide a rationale for prospective studies evaluating stratified pleural intervention pathways based on disease responsiveness and predicted pleural trajectory.

## 5. Conclusions

In this retrospective cohort of patients with haematological malignancy-associated malignant pleural effusion, the majority of patients initially managed with an aspiration-first strategy avoided definitive pleural intervention. In contrast to solid tumour-associated MPE, many effusions resolved following systemic therapy alone, likely reflecting the chemo-responsive nature of the underlying malignancy. Non-chemo-responsive disease was associated with earlier progression to definitive pleural intervention. These findings suggest that routine extrapolation of solid tumour-derived pleural management paradigms may risk over-intervention in selected patients with haematological malignancy and support a more individualised approach to pleural management in this population.

## Figures and Tables

**Table 1:** Baseline characteristics of patients with haematological malignancy-associated malignant pleural effusion.

Characteristic	Overall Cohort (*n* = 116)
Age, median (IQR)	68 (60–76)
Male sex, *n* (%)	68 (59%)
Female sex, *n* (%)	48 (41%)
Lymphoma, *n* (%)	70 (60%)
Plasma cell dyscrasia, *n* (%)	24 (21%)
Leukaemia, *n* (%)	14 (12%)
Other haematological malignancy, *n* (%)	8 (7%)
Septated/complex pleural effusion, *n* (%)	32 (28%)
Chemo-responsive disease, *n* (%)	74 (64%)
Non-chemo-responsive disease, *n* (%)	42 (36%)

## Data Availability

The datasets generated and/or analysed during the current study are not publicly available because they contain potentially identifiable patient information. De-identified data may be made available from the corresponding author on reasonable request, subject to approval by the sponsoring institution and applicable governance and data protection requirements.
